# Translating DNA into Synthetic Molecules

**DOI:** 10.1371/journal.pbio.0020223

**Published:** 2004-07-13

**Authors:** David R Liu

## Abstract

The unique ability of nucleic acids to replicate has recently been combined with the power of combinatorial chemistry, providing a new approach to the science of drug design

At some time almost 4 billion years ago, nature likely was faced with a chemical dilemma. Nucleic acids had emerged as replicable information carriers and primitive catalysts (Joyce 2002), yet their functional potential was constrained by their structural homogeneity and lack of reactive groups. These properties rendered nucleic acids well suited for storing information, but flawed for mediating the diverse chemistries required to sustain and improve increasingly complex biological systems. It is tempting to speculate that translation emerged as the solution to this dilemma. Translation, defined here as the conversion of an informationcarrying molecule into a corresponding encoded structure, enabled the expanded functional potential of proteins to be explored using powerful evolutionary methods that depend on the unique ability of nucleic acids to replicate.

A small but growing number of researchers have begun to tackle a modern version of this dilemma. While proteins and nucleic acids can be manipulated using powerful molecular biology techniques that enable their directed evolution, the size, fragility, and relatively limited functional group diversity of biological macromolecules make them poorly suited for solving many problems in the chemical sciences. Ideally, researchers would like to apply evolution-based approaches to the discovery of functional synthetic, rather than biological, molecules. A solution analogous to nature's translation of mRNA into protein could, in principle, address this contemporary problem (Orgel 1995; Gartner and Liu 2001). If a laboratory system were developed that could translate amplifiable information carriers such as DNA into arbitrary synthetic molecules, the evolution of synthetic molecules using iterated cycles of translation, selection, amplification, and diversification would be possible.

The translation of DNA into synthetic molecules is conceptually distinct from the use of DNA simply as a tag during the solidphase synthesis of a molecule that is part of a combinatorial library (Brenner and Lerner 1992). The latter process uses DNA to record the history of a series of chemical reactions by cosynthesizing a portion of a DNA oligonucleotide during each step of a molecule's solidphase synthesis. As a result, the identity of compounds that pass screening can be inferred by PCR amplification and sequencing of the DNA associated with a given bead (Needels et al. 1993). The resulting DNA, however, cannot redirect the synthesis of active compounds. In contrast, the translation of DNA into synthetic molecules uses the sequence of nucleotides in a strand of DNA to direct the synthesis of a nascent molecule. As a result, a complete cycle of translation, selection, and amplification can be applied to the discovery of synthetic molecules in a manner that is analogous to the processes that take place during biological evolution.

DNA-templated organic synthesis (DTS) has emerged as one way to translate DNA sequences into a variety of complex synthetic small molecules (Gartner and Liu 2001; Gartner et al. 2002; Li and Liu 2004). In this approach, starting materials covalently linked to DNA templates approximately 20–50 nucleotides in length are combined in very dilute solutions with reagents that are covalently linked to complementary DNA oligonucleotides. Upon Watson-Crick base pairing, the proximity of the synthetic reactive groups elevates their effective molarity by several orders of magnitude, inducing a chemical reaction. Because reactions do not take place between reactants linked to mismatched (noncomplementary) DNA, DTS generates synthetic products in a manner that is programmed by the sequence of bases in the template strand.

In a series of three papers in this issue of *PLoS Biology*, Harbury and co-workers describe an elegant new approach to translating DNA into synthetic peptides called “DNA display.” Their approach uses DNA hybridization to separate mixtures of DNA sequences into spatially distinct locations. The first paper (Halpin and Harbury 2004a) reports the development of resin-linked oligonucleotides that efficiently and sequence-specifically capture DNA containing complementary subsequences. This immobilization process is efficient enough to be iterated, so that DNA sequences specifying multiple amino acids can be routed to the appropriate miniature resin-filled columns during each step.

In the second paper (Halpin and Harbury 2004b), Harbury and coworkers detail solid-phase peptide synthesis performed on unprotected DNA 340mers bound to DEAE Sepharose. Optimization of amino acid side-chain-protecting groups and peptide coupling conditions enabled a variety of amino acids to undergo efficient peptide coupling to bound oligonucleotides containing an amine group.

The third paper (Halpin et al. 2004) integrates the routing and peptide synthesis described above into the translation of a library of 10^6^ DNA 340mers into a corresponding library of up to 10^6^ synthetic pentapeptides. To achieve chemical translation, the DNA library was subjected to iterated cycles of routing and solidphase peptide synthesis. After each routing step, the appropriate amino acid was coupled to each DNA-linked subpopulation. DNA routing was therefore used to achieve the splitting step of “split-and-pool” combinatorial peptide synthesis. The completed library of peptide–DNA conjugates was then subjected to in vitro selection based on the ability to bind an antibody with known affinity for the [Leu]enkephalin pentapeptide Tyr-Gly-Gly-Phe-Leu. After two rounds of routing, synthesis, and selection, followed by DNA sequencing, the remaining oligonucleotides predominantly encoded the Tyr-Gly-Gly-Phe-Leu sequence or close variants thereof. This result demonstrates that the DNA display method is capable of facilitating the discovery of functional molecules by enabling in vitro selection methods to be applied to molecules generated by split-and-pool combinatorial synthesis.

The fundamental distinctions between DTS and DNA display approaches to chemical translation imply that these two strategies will be applicable to different types of synthetic structures. Because the DNA display approach separates the DNA hybridization step from the chemical synthesis step, it does not require the coupling of synthetic reagents to oligonucleotides (beyond the starting material), and can use reaction conditions such as high temperatures or high pH that may not be compatible with DNA hybridization. These features suggest that DNA display may be able to access structures that cannot be created by DTS. Likewise, because DTS approaches use effective molarity rather than intermolecular reactivity to direct organic synthesis, they enable modes of controlling reactivity (such as using otherwise incompatible reactions in a single solution [Calderone et al. 2002]) and classes of chemical reactions (such as heterocoupling of substrates that preferentially homocouple) that cannot be accessed using split-and-pool synthesis. In principle, these two approaches are complementary, and it is tantalizing to envision the use of both DNA display and DTS to direct different steps during a single chemical translation.

In order for either approach to fully realize its potential of truly *evolving* libraries of diverse synthetic molecules, rather than simply enriching libraries that already contain at the outset the “most fit” molecule, researchers must develop sophisticated library syntheses that generate remarkable complexity (vast numbers of different compounds) in a relatively modest number of DNA-compatible synthetic steps. True evolution takes place when the theoretical complexity of a population exceeds the number of different molecules that can be created in a single library translation step, and when diversification is required to access compounds in later generations that are more fit than any member of the starting pool.

To my knowledge, no synthetic library to date contains this degree of complexity (indeed, the total size of the Chemical Abstracts Service database of known chemical substances is presently less than 10^8^ compounds). However, because so few copies of a DNA-linked synthetic molecule are required for in vitro selection (Doyon et al. 2003)—compared with the relatively large quantities of material that are required for conventional screening approaches—these chemical translation methods offer the first hope of achieving such synthetic complexity without requiring an impractical amount of material or storage space. For comparison, a conventional-format synthetic library containing 100 µg of each of 10^8^ different structures would represent 10 kg of material, not including the mass of beads or plates associated with the library, while a chemically translated library containing 10,000 copies of 10^8^ different species represents less than 1 µg of total material.

While significant remaining challenges face efforts to develop and apply chemical translation, the promise of marrying evolution and organic synthesis is an irresistible combination for some researchers. The work of Harbury and co-workers described in this issue represents the latest approach to the very ancient problem of translating replicable information into functional structures.

## Abbreviation

DTS: DNA-templated organic synthesis
